# HSP60 Regulates Lipid Metabolism in Human Ovarian Cancer

**DOI:** 10.1155/2021/6610529

**Published:** 2021-09-12

**Authors:** Na Li, Nannan Li, Siqi Wen, Biao Li, Yaying Zhang, Qing Liu, Shu Zheng, Jingru Yang, Liang Shen, Ligang Xing, Xianquan Zhan

**Affiliations:** ^1^Shandong Key Laboratory of Radiation Oncology, Cancer Hospital of Shandong First Medical University, 440 Jiyan Road, Jinan, Shandong 250117, China; ^2^Medical Science and Technology Innovation Center, Shandong First Medical University, 6699 Qingdao Road, Jinan, Shandong 250117, China; ^3^Key Laboratory of Cancer Proteomics of Chinese Ministry of Health, Xiangya Hospital, Central South University, 87 Xiangya Road, Changsha, Hunan 410008, China; ^4^Department of Gynecology, Shandong Provincial Hospital Affiliated to Shandong First Medical University, 324 Jingwu Weiqi Road, Jinan, Shandong 250021, China

## Abstract

Accumulating evidence demonstrates that cancer is an oxidative stress-related disease, and oxidative stress is closely linked with heat shock proteins (HSPs). Lipid oxidative stress is derived from lipid metabolism dysregulation that is closely associated with the development and progression of malignancies. This study sought to investigate regulatory roles of HSPs in fatty acid metabolism abnormality in ovarian cancer. Pathway network analysis of 5115 mitochondrial expressed proteins in ovarian cancer revealed various lipid metabolism pathway alterations, including fatty acid degradation, fatty acid metabolism, butanoate metabolism, and propanoate metabolism. HSP60 regulated the expressions of lipid metabolism proteins in these lipid metabolism pathways, including ADH5, ECHS1, EHHADH, HIBCH, SREBP1, ACC1, and ALDH2. Further, interfering HSP60 expression inhibited migration, proliferation, and cell cycle and induced apoptosis of ovarian cancer cells *in vitro*. In addition, mitochondrial phosphoproteomics and immunoprecipitation-western blot experiments identified and confirmed that phosphorylation occurred at residue Ser70 in protein HSP60, which might regulate protein folding of ALDH2 and ACADS in ovarian cancers. These findings clearly demonstrated that lipid metabolism abnormality occurred in oxidative stress-related ovarian cancer and that HSP60 and its phosphorylation might regulate this lipid metabolism abnormality in ovarian cancer. It opens a novel vision in the lipid metabolism reprogramming in human ovarian cancer.

## 1. Introduction

In a biological context, free radical reactive oxygen species (ROS) is a normal byproduct of oxygen metabolism, which plays positive roles in cell signaling and homeostasis [1]. However, the dramatically increased ROS levels are related to numerous diseases and degenerative processes, such as autoimmune diseases, cancers, and Alzheimer's diseases [2]. Mitochondria were one of the crucial places to produce ROS during the oxidative phosphorylation process. When electrons are passed through the electron transport chain via oxidation-reduction reactions across the inner mitochondrial membrane, a few oxygen is incompletely reduced to give the superoxide radical (^∙^O_2_^−^) through a series of proteins, such as complex I and complex III of mitochondria [3]. In general, the superfluous ROS would have harmful effects on the cells via damage of DNA or RNA, oxidation of amino acids, damage of mitochondrial and cytoskeletal functions, lipid peroxidation, and oxidative deactivation of specific enzymes [4]. Cancer cells showed greater ROS stress compared to normal cells, partly due to increased metabolic activity, carcinogenesis stimulation, and mitochondrial malfunction [5]. In ovarian cancer, the previous studies showed that ROS could activate various oncogenic transcription factors such as NF-*κ*B [6], AP-1 [7], and STAT3 [8], to influence cellular transformation, tumor cell proliferation, inflammation, tumor cell survival, metastasis and invasion, and angiogenesis. Moreover, molecular and cellular function analysis of ovarian cancer mitochondrial expressed proteins (mtEPs) found that many mitochondrial differentially expressed proteins (mtDEPs) were associated with ROS production, such as generation of active oxygen species, metabolism of hydrogen peroxide, and synthesis, metabolism, production, and quantity of ROS [9]. It clearly demonstrated that ovarian cancer was an ROS-related disease or oxidative stress-related disease.

Heat shock proteins (HSPs), also known as stress proteins, were a conserved mitochondrial chaperone family. The abnormal posttranslational modifications (PTMs), abundance levels, cellular localization, or mutations of HSPs would influence multiple cellular biological processes [10]. HSPs played a key role in pathophysiology and pharmacology of cancer, which was leading to cancer growth, drug resistance, local infiltration, and distant metastasis. HSPs were proven to be potential clinical biomarkers for cancer early diagnosis, follow-up surveillance, and therapeutic targets in multiple cancers [11]. HSP60, as one classical member of the HSP family, was a crucial mitochondrial chaperone and involved in the protein folding process that converts an unfolded substrate into a folded substrate to the mitochondrial matrix [12]. The domain of HSP60 protein was similar to its prokaryotic ortholog GroEL, which indicated the mechanism of HSP60 protein in cancer. In short, unfolded proteins bound to the amino acid residue of HSP60 cavity. Also, HSP60 interacted with another chaperone HSP10 and ATP, which induced conformational changes of HSP60 to change the properties of the binding sites—in turn opening or closing the HSP60 cavity. After the substrate protein completed protein folding and obtained its native conformation in several cycles, it would be released [13]. Therefore, HSP60 had a pivotal role in the misfolded processes of protein homeostasis that was associated with various diseases. Additionally, ROS was also typically seen in diseases associated with misfolded proteins and had been reported to result in apoptosis [14]. HSP60 was proven to be a mediator of ROS [15], and ROS in turn could also regulate the expression and PTMs of the HSP family [16]. For example, proteomic analysis found that HSP60 silencing increased excessive ROS and drove metabolic reprogramming to promote cell growth and tumor progression via activating NRF2-mediated oxidative stress responses [15]. ROS was also reported to regulate HSPs via the JAK/STAT pathway. Mechanistically, H_2_O_2_ induced expressions of HSP70 in a time-dependent manner by activating the HSP70 promoter with enhanced STAT binding [16]. Even though the relationship between oxidative stress and HSPs has been demonstrated in numerous studies, the molecular mechanism and the downstream signaling pathways of HSPs remain unclear.

Metabolic alterations have developed into a new hallmark in cancer researches. Evidence showed that expressions of hub molecules were changed in the lipid metabolism pathway, which raised the idea that lipid biomarkers might provide broad prospects for cancer diagnosis and that inhibition of the lipid metabolism process in cancer cells might hold therapeutic implications [17]. A variety of lipid molecules and their metabolic intermediates might participate in inflammation, cell signal transduction, cell adhesion and movement, angiogenesis, and cell proliferation. Abnormal metabolism in cancer cells had the characteristics of an uncontrolled de nova fatty acid synthesis pathway; thus, the increased lipid synthesis and decreased fatty acid degradation could generate a relatively high proliferation rate [18]. One study showed that upregulation of lipogenesis pathways was associated with poor survival outcomes in cancer, and lipid metabolism was closely related to the malignant phenotype (invasion, migration, etc.) of tumor cells in *in vitro* experiments [19]. Hyperlipidemia promoted hormone-related cancers (ovarian cancer, endometrial cancer, etc.). Therefore, an abnormal blood fat index might be a useful marker to evaluate some types of tumors. Cancer cells also produced a series of lipolysis substances and released them into the blood, such as TNF-*α*, glucocorticoid, hormone-sensitive lipase (HSL), and lipid-mobilizing factor (LMF/ZAG) [20]. HSL was detectable in serum and ascites of patients with ovarian cancer, and the activity of HSL was significantly higher (3 times) than that of the normal control based on clinical data [21]. Additionally, lipids would transfer from adipocytes to ovarian cancer cells by coculture condition, which promoted cancer cell growth [22]. All the evidence suggested that lipid disorder was closely related to tumorigenesis and tumor development. Novel applications of electrospray ionization mass spectrometry (ESI/MS) facilitated lipidomic development [23]. Lipidomics, as a novel subject, revealed altered biosynthetic pathways in various vital phenomena and cell signaling as biomarkers. Lipid metabolism disorders or reprogramming of tumors formed tumor lipidomics, which could provide potential biomarkers and therapeutic targets for cancer patients. Lipid metabolism dysregulation, as an important part of energy reprogramming, was closely associated with the development and progression in cancers. Moreover, KEGG pathway network analysis of ovarian cancer mtEPs (*n* = 5115) revealed that many mtEPs were significantly involved in lipid metabolism pathways, including fatty acid degradation, fatty acid metabolism, butanoate metabolism, and propanoate metabolism [24]. Ingenuity pathway analysis (IPA) of ovarian cancer mtDEPs (*n* = 1198) also revealed that many mtDEPs were also significantly involved in lipid metabolism pathways, including fatty acid beta-oxidation I [9]. Lipid oxidative stress was derived from lipid metabolism dysregulation that was closely associated with the development and progression of malignancies. However, the regulatory mechanisms of fatty acid metabolism abnormality in ovarian cancer remain unclear.

The previous studies demonstrated that HSP60 knockdown drove metabolic reprogramming in clear cell renal cell carcinoma to promote tumor progression and enhance mitochondrial-dependent biosynthesis [25]. In terms of lipid metabolic reprogramming, one study indicated that HSP60 might be involved in the lipid regulatory effect of whole grain highland hull-less barley (WHLB). Quantitative real-time polymerase chain reaction analysis showed that rats given with WHLB diet exhibited downregulated expression of HSP60 compared to rats given with the control high-fat diet [26]. The relationship between lipid hydroperoxide (LOOH) level and Hsp60 was investigated in sera from newborns, which proved that Hsp60 level was positively correlated with LOOH level [27]. In terms of regulatory mechanisms between HSP60 and lipid metabolism, one study demonstrated that the HSP10-HSP60 chaperoning protein folding complex played a role in the folding of medium-chain acyl-CoA dehydrogenase (MCAD), and this folding process was influenced by deacetylation of HSP10 [28]. In HEK-293 cells, both the amounts of total MCAD proteins and folded MCAD proteins were closely related to the expression levels of HSP60. HSP60 downregulation impaired both wild-type and disease-associated MCAD proteins. A similar result was observed that the mitochondrial short-chain acyl-CoA dehydrogenase (ACADS) wild type was also regulated by the HSP60/10 folding machinery [29]. Acyl-CoA dehydrogenases were important in multiple lipid metabolism pathways, and those previous researches demonstrated that HSP60/10 chaperoning proteins were crucial for the folding process of acyl-CoA dehydrogenase. Dysregulation of HSP60 expression levels did jeopardize those kinds of protein folding, leading to the alteration of fatty acid oxidation and energy supply. Additionally, a previous phosphoproteomic study found a new phosphorylation site at residue Ser70 in HSP60 in mitochondrial samples from ovarian cancer tissues, and its phosphorylation level was significantly increased in ovarian cancers compared to controls [30]. These previous findings clearly revealed the potential functions of HSP60 for mitochondrial protein folding related to lipid metabolism pathways, which further showed the roles of HSP60 in cancer development.

**Figure 1:**
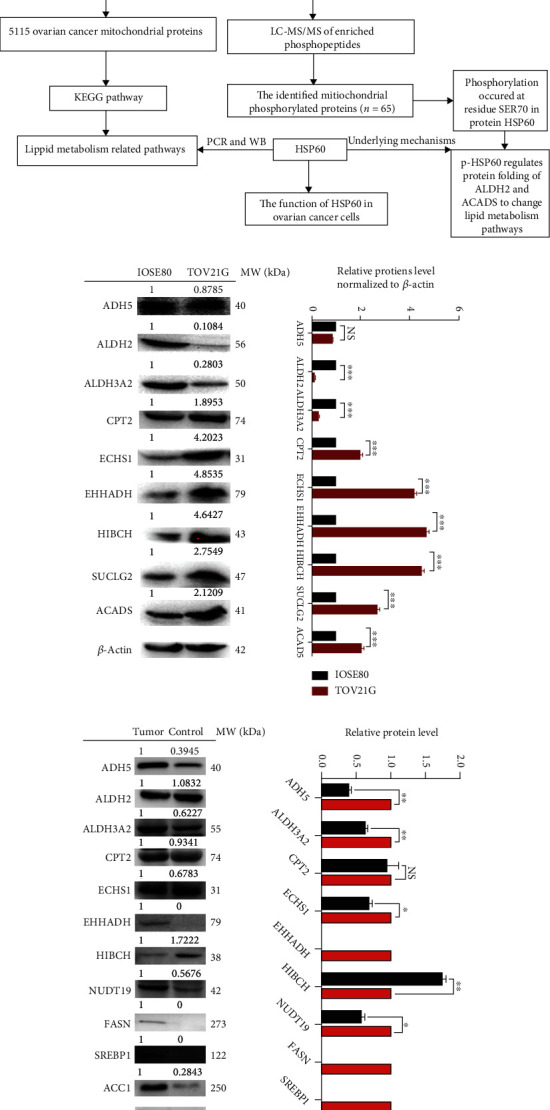
Experimental flowchart and verifications of mtDEPs in lipid metabolism pathways. (a) Experimental flowchart to identify lipid metabolism-related pathways and underlying mechanisms. (b) Western blot analysis of ADH5, ALDH2, ALDH3A2, CPT2, ECHS1, EHHADH, HIBCH, SUCLG2, and ACADS in ovarian cancer cells TOV21G compared to control cells IOSE80. (c) Western blot analysis of ADH5, ALDH2, ALDH3A2, CPT2, ECHS1, EHHADH, HIBCH, NUDT9, FASN, ACC1, and SREBP1 in lipid metabolism pathways between ovarian cancer tissues and benign ovarian disease tissues. *n* = 3. ^∗∗∗^*p* < 0.001. NS: no significance.

This present study used the integrative analysis of mtEP (*n* = 5115) and mitochondrial phosphoprotein (mtPP) datasets in ovarian cancers, which found that HSP60 might be related to lipid metabolism pathways in ROS-related ovarian cancers. The multiple cellular lipid metabolism signaling pathways were changed based on the mtEP dataset, which might play an important role in ovarian cancer invasion and metastasis. In addition, functional analyses demonstrated that HSP60 knockdown suppressed cell proliferation and migration and induced apoptosis and cell cycle arrest. Also, mitochondrial HSP60 was differentially phosphorylated at its residue Ser70. HSP60 was highly related to lipid metabolism and might regulate fatty acid oxidation by influencing both the expression and folding of related enzymes in the lipid metabolism pathways, and regulatory roles of phosphorylated HSP60 in lipid metabolism pathways were further investigated in ovarian cancers. The experimental flowchart is shown in Figure 1(a).

## 2. Material and Methods

### 2.1. Ovarian Cancer Mitochondrial Protein Dataset

In total, 5115 ovarian cancer mtEPs were identified by isobaric tags for relative and absolute quantitation- (iTRAQ-) based quantitative proteomics [24], including 1198 mtDEPs in human epithelial ovarian cancers relative to control ovary tissues [9].

### 2.2. Ovarian Cancer Phosphoprotein Dataset

In total, 67 ovarian cancer mtPPs were identified by TiO_2_ enrichment-based iTRAQ quantitative phosphoproteomics [30].

### 2.3. Pathway Network Analysis

The IPA software (https://digitalinsights.qiagen.com/products-overview/discovery-insights-portfolio/analysis-and-visualization/qiagen-ipa/) was used to analyze 1198 mtDEPs for determining their molecular and cellular functions in ovarian cancers. Kyoto Encyclopedia of Genes and Genomes (KEGG) pathways with DAVID Bioinformatics Resources 6.7 (https://david.ncifcrf.gov/home.jsp) were used to analyze 5115 mtEPs for determining signaling pathway networks in ovarian cancers. STRING 10.0 (http://string-db.org/cgi/input.pl) was used to construct the protein-protein interaction between the proteins in the identified lipid metabolism pathways and HSP60. PYMOL (http://www.pymol.org) was used to modularize the structures of human HSP60-ACADS and HSP60-ALDH2. Protein kinase-specific prediction (http://bioinfo.ncu.edu.cn/PKPred_Prediction.aspx) was used to analyze the upstream kinase of HSP60 (Ser70) based on a single kinase.

### 2.4. Western Blot Verification of mtDEPs in Lipid Metabolism Pathways in the Ovarian Cancer Cell Line

Western blot was used to verify each mtDEP in lipid metabolism pathways, including ADH5, ALDH2, ALDH3A2, CPT2, ECHS1, EHHADH, HIBCH, SUCLG2, and ACADS (BBI Life Science Corporation, HK, China), between the ovarian cancer cell TOV-21G and the normal ovarian epithelial cell IOSE80. Additionally, western blot was used to verify each mtDEP in lipid metabolism pathways, including ADH5, ALDH2, ALDH3A2, CPT2, ECHS1, EHHADH, HIBCH, NUDT9, FASN, ACC1, and SREBP1 (Abcam, UK), between ovarian cancer tissues and control ovary tissues. *β*-Actin was used as the internal standard.

### 2.5. Effects of HSP60 on Ovarian Cancer Cell Biological Behaviors

Transient transfection of the ovarian cancer cell TOV-21G was used in this study. Cells TOV-21G and IOSE80 were ordered from the Keibai Academy of Science (Nanjing, China). TOV-21G cells were cultured in 1640 medium (Thermo Fisher Scientific, USA) containing 10% fetal bovine serum (Biological Industries, South America). IOSE80 cells were cultured in DMEM (Thermo Fisher Scientific, USA) containing 10% fetal bovine serum (Biological Industries, South America). The si­HSP60 and si­control were designed and synthesized by GenePharma (Shanghai, China). The si-RNA sequences for HSP60 were 5′-GACGAUGCCAUGCUCUUAATT-3′ (sense strand) and 5′-UUAAGAGCAUGGCAUCGUCTT-3′ (antisense strand). TOV-21G cells (2 × 10^5^) were seeded in 6-well plates, and transient transfection was performed when cell density is up to 30-50%. TOV-21G cells in different wells were transfected with si­HSP60 (20 *μ*M) or si-control using Lipofectamine 3000 reagents (Invitrogen, USA), followed by incubation for 24-48 h. Then, the following experiments were done: (i) a wound healing assay was used to detect cell mobility between control and si-Hsp60 groups. TOV-21G cells were photographed when split with a micropipette tip (10 *μ*l) and 36 h after being split. (ii) A CCK8 cell assay was used to detect cell proliferation of TOV-21G cells between control and si-HSP60 groups. (iii) An EdU assay was used to measure positive proliferative cells with fluorescence staining DNA synthesis between control and si-HSP60 groups. (iv) An apoptosis assay was performed with an annexin V-fluorescein isothiocyanate (FITC) apoptosis detection kit (BestBio, Shanghai). Briefly, TOV-21G cells (2 × 10^6^) in si-HSP60 and control groups were washed 3 times with PBS. Annexin V-FITC and propidium iodide (PI) were added at room temperature away from light and incubated for 20 min. Fluorescence-activated cell sorting (FACS) using a BD Accuri™ C6 (BD Biosciences, Ashland, OR, USA) was performed, and the apoptosis rate was analyzed with FlowJo (Tree Star, Ashland, OR, USA). (v) Cells (2 × 10^6^) were washed 3 times with PBS and fixed with absolute ethyl alcohol (-20°C, 2 h). After washing with PBS, cells were coincubated with PI and 20 *μ*l RNaseA (BestBio, Shanghai) at 37°C in a water bath for 30 min away from light. Cells were then analyzed for DNA proportion distribution with FACS in a BD Accuri™ C6 (BD Biosciences), and then, the cell cycle was analyzed with ModFit LT for Windows version 3.2. Extrinsic and intrinsic apoptosis evaluation was necessary for a better understanding of the apoptotic pathway activated in HSP-silenced cancer cells. Western blot was used to verify apoptosis-related proteins, including caspase-3, caspase-8, and caspase-9 between si-HSP60 and control groups.

### 2.6. qRT-PCR and Western Blot Were Used to Verify HSP60-Targeted Molecules in Lipid Metabolism Pathways

Total RNAs were isolated from three biological replicates of si-HSP60 or control groups with the TRizol reagent (Invitrogen). The mRNAs from 500 ng of total RNAs were reversely transcribed into cDNAs for quantitative real-time PCR (qRT-PCR) analysis with the SYBR Premix ExTaq kit (TaKaRa). Beta-actin was set as an internal control for gene quantification. Supplementary Table 1 contains those mRNA molecules and their corresponding primers for qRT-PCR analysis, including ADH5, ALDH2, ALDH3A2, CPT2, ECHS1, EHHADH, HIBCH, SUCLG2, and ACADS. Western blot was used to verify apoptosis-related proteins, including ADH5, ALDH3A2, CTP2, ECHS1, EHHADH, HIBCH, NUDT19, FASN, SREBP1, ACC1, ACADS, and ALDH2 in TOV-21G cells between si-HSP60 and control groups.

### 2.7. Validation of Phosphorylated HSP60 Using Immunoprecipitation

An anti-phospho-HSP60 (Ser_70_) antibody was prepared by Affinity Company (USA), and the polypeptide synthesis sequence of HSP60 was tVIIEQSWGsPK. The brief procedure was as follows: (i) polypeptide with phosphorylation at residue Ser70 in HSP60 was synthesized (12-16 amino acids, purity greater than 90%, and 10 mg), without phosphorylation modification in the other amino acid residues. (ii) KLH was coupled with cysteine of C or N terminal. (iii) Two New Zealand rabbits were immunized, and peripheral blood was tested after ten weeks. (iv) Antigen was purified by affinity chromatography for two steps to obtain 5 mg anti-phospho-HSP60 (Ser_70_) antibody and 1 mg anti-HSP60 antibody. (v) Mass spectrometry and ELISA were used to detect the quality of affinity-synthesized antigen.

Phosphorylated HSP60 (Ser70) between ovarian cancer cells TOV-21G and control IOSE80 was semiquantified by immunoprecipitation (IP) and western blot. Briefly, HSP60 proteins were immunoprecipitated with a mouse anti-human HSP60 monoclonal antibody (10 *μ*g; Proteintech, USA) or a mouse anti-human IgG monoclonal antibody (5 *μ*g; Proteintech, USA) from an amount (1 mg) of proteins extracted from the ovarian cancer cell TOV-21G and control cell IOSE80. The total proteins and IP products of the ovarian cancer cell TOV-21G and control cell IOSE80 were used to detect phosphorylated HSP60 (Ser70) by western blotting based on a rabbit anti-human phosphorylation antibody (1 : 500 dilution).

### 2.8. Statistical Analysis

For KEGG enrichment analysis and IPA pathway network analysis, *p* values were corrected by Benjamini-Hochberg multiple testing. For western blot, wound healing assay, CCK8 cell assay, EdU assay, apoptosis assay, cell cycle assay, and qRT-PCR data, data were expressed as mean ± SD with a statistically significant level of *p* < 0.05 using the Student *t*-test in SPSS 13.0 (SPSS Inc., Chicago, USA) (*n* = 3).

## 3. Results

### 3.1. KEGG Pathway Analysis Revealed Lipid Metabolism Pathway Alterations in Ovarian Cancers

KEGG pathway analysis of 5115 mtEPs in ovarian cancers revealed various lipid metabolism pathways, including fatty acid degradation, fatty acid metabolism, butanoate metabolism, and propanoate metabolism [24] (Table 1; Supplementary Figure 1). Fatty acid degradation-related proteins were significantly increased in ovarian cancers relative to controls (fold change > 1.5), including CPT2 (fold change = 2.05, *p* = 1.99*E* − 2), ACOX1 (fold change = 1.53, p =3.40E-2), EHHADH (fold change = 1.62, *p* = 2.25*E* − 3), ECHS1 (fold change = 1.52, *p* = 3.56*E* − 3), and ECI1 (fold change = 1.64, *p* = 1.99*E* − 3). Fatty acid metabolism-related proteins were significantly increased in ovarian cancers relative to controls (fold change > 1.5), including ACAA1 (fold change = 2.70, *p* = 1.00*E* − 2), ACOX1 (fold change = 1.53, *p* = 3.40*E* − 2), CPT2 (fold change = 2.05, *p* = 1.99*E* − 2), ECHS1 (fold change = 1.52, *p* = 3.56*E* − 3), ECI1 (fold change = 1.64, *p* = 1.99*E* − 3), and PECR (fold change = 1.57, *p* = 4.14*E* − 4). Butanoate metabolism-related proteins were significantly increased in ovarian cancers relative to controls (fold change > 1.5), including EHHADH (fold change = 1.62, *p* = 2.25*E* − 3), ECHS1 (fold change = 1.52, *p* = 3.56*E* − 3), BDH1 (fold change = 1.54, *p* = 3.00*E* − 3), and HMGCS2 (fold change = 2.17, *p* = 2.10*E* − 3). Propanoate metabolism-related proteins were significantly increased in ovarian cancers relative to controls (fold change > 1.5), including GSTK1 (fold change = 2.13, *p* = 4.20*E* − 5), ACAA1 (fold change = 2.70, *p* = 1.00*E* − 2), NUDT19 (fold change = 1.55, *p* = 6.40*E* − 3), PECR (fold change = 1.57, *p* = 4.14*E* − 4), PEX13 (fold change = 1.61, *p* = 3.58*E* − 3), PEX14 (fold change = 1.99, *p* = 6.51*E* − 4), IDH2 (fold change = 2.02, *p* = 2.07*E* − 3), ABCD3 (fold change = 1.99, *p* = 6.51*E* − 4), and PHYH (fold change = 1.94, *p* = 7.71*E* − 3).

To validate mtDEPs in these lipid metabolism pathways derived from iTRAQ quantitative proteomics, mtDEPs were further examined by western blotting in ovarian cancer cells TOV21G and control cells IOSE80 (Figure 1(b)), including ADH5 (fold change = 0.85, *p* = 0.0682), ALDH2 (fold change = 0.12, *p* < 0.0001), and ALDH3A2 (fold change = 0.30, *p* < 0.0001) enriched in the fatty acid degradation pathway; CPT2 (fold change = 1.99, *p* < 0.0001) enriched in fatty acid degradation and fatty acid metabolism pathways; ECHS1 (fold change = 4.15, *p* < 0.0001) enriched in fatty acid degradation, butanoate metabolism, and propanoate metabolism pathways; EHHADH (fold change = 4.67, *p* < 0.0001) enriched in fatty acid degradation, fatty acid metabolism, butanoate metabolism, and propanoate metabolism pathways; HIBCH (fold change = 4.48, *p* < 0.0001) and SUCLG2 (fold change = 2.68, *p* < 0.0001) enriched in the propanoate metabolism pathway; and ACADS (fold change = 2.04, *p* < 0.0001) enriched in fatty acid degradation, fatty acid metabolism, and butanoate metabolism pathways. These western blotting results in cell models had a good consistency with the results of iTRAQ quantitative mitochondrial proteomics in ovarian cancer tissues.

Moreover, mtDEPs were also examined by western blotting between ovarian cancer tissues and normal control tissues (Figure 1(c)), including ADH5, ALDH2, ALDH3A2, CPT2, ECHS1, EHHADH, HIBCH, and NUDT9 enriched in metabolism pathways. Further, the key enzymes of fatty acid synthesis metabolism were significantly expressed between ovarian cancer tissues and normal control tissues, including FASN, ACC1, and SREBP1. The most of these western blotting results in tissues had a good consistency with the results of iTRAQ quantitative mitochondrial proteomics in ovarian cancer tissues. It indicated that lipid metabolism might be disordered in ovarian cancer.

### 3.2. si-HSP60 Inhibited *In Vitro* Migration and Proliferation of Ovarian Cancer Cells

**Figure 2:**
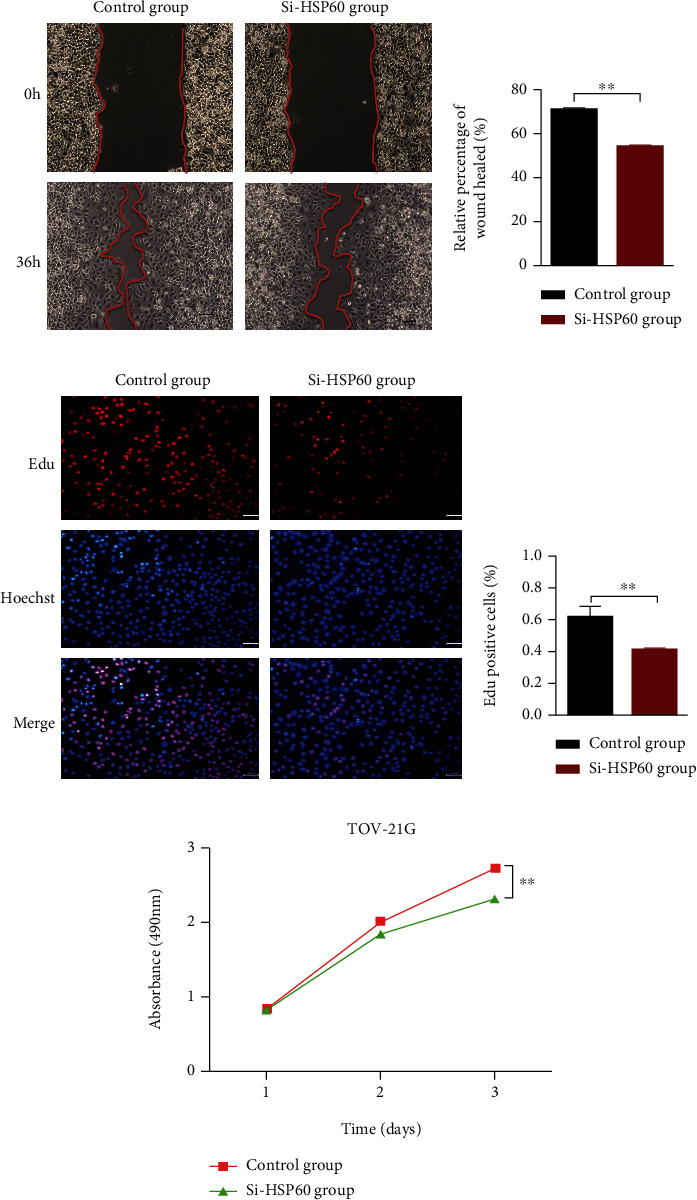
The si-HSP60 inhibited the migration and proliferation of ovarian cancer cells *in vitro*. (a) Cell migration was measured by the wound healing assay in TOV-21G cells transfected with si-HSP60 and control sequences (*n* = 3). (b) The histogram of cell migration results with the wound healing assay in TOV-21G cells transfected with si-HSP60 and control sequences (*n* = 3). (c) EdU cell proliferation test of TOV-21G transfected with si-HSP60 and control sequences (*n* = 3). (d) The histogram of EdU cell proliferation test of TOV-21G transfected with si-HSP60 and control sequences (*n* = 3). (e) CCK8 cell proliferation test of TOV-21G transfected with si-HSP60 and control sequences (*n* = 3). ^∗^*p* < 0.05, ^∗∗^*p* < 0.01, and ^∗∗∗^*p* < 0.001.

The knockdown of HSP60 was established in the TOV-21G cell line model with more than 60% inhibition rate at the inhibition level. The anticancer ability of si-HSP60 was measured with the wound healing assay, CCK8 assay, and EdU assay between si-HSP60-transfected and control TOV-21G cell groups. Downregulated expression of HSP60 significantly slowed wound healing (Figures 2(a) and 2(b)), decreased the number of EdU-positive cells (Figures 2(c) and 2(d)), and inhibited ovarian cancer cell growth (Figure 2(e)), when compared to the control group. These results clearly demonstrated that si-HSP60 significantly inhibited *in vitro* migration and proliferation of ovarian cancer cells.

### 3.3. si-HSP60 Inhibited Cell Cycle Progression and Promoted Cell Apoptosis of Ovarian Cancer Cells

**Figure 3:**
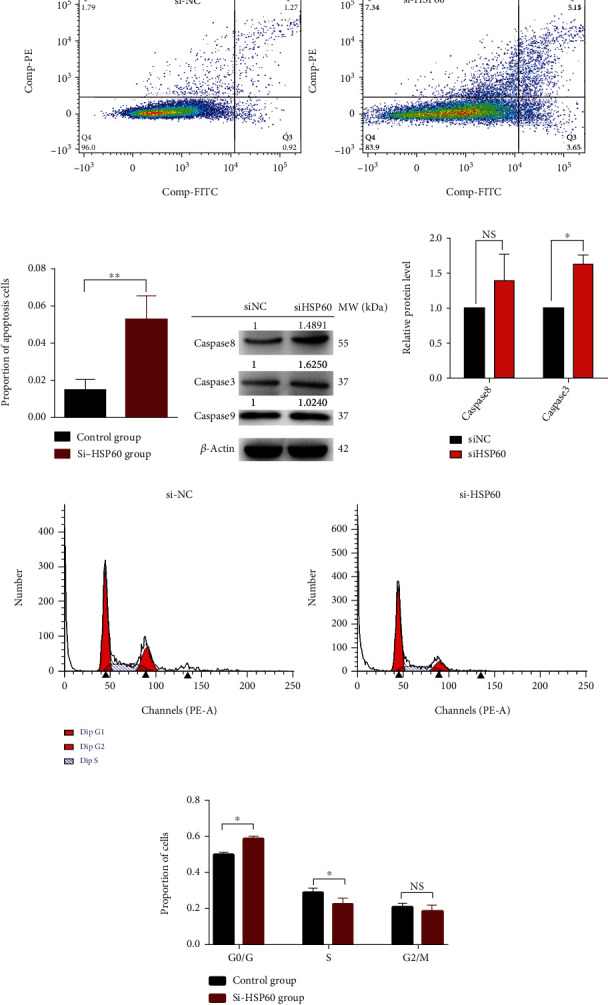
The si-HSP60 promoted apoptosis and inhibited the cell cycle of ovarian cancer cells *in vitro*. (a) Apoptosis percentage of TOV-21G cells transfected with si-HSP60 and control sequences, measured by fluorescence-activated cell sorting (FACS) (*n* = 3). (b) The histogram of apoptosis percentage of TOV-21G cells transfected with si-HSP60 and control sequences, measured by fluorescence-activated cell sorting (FACS) (*n* = 3). (c) Western blots for caspase-3, caspase-8, and caspase-9 to evaluate extrinsic and intrinsic apoptosis pathways. (d) The histogram of caspase-3 and caspase-8 expression between si-HSP60 and control sequence groups. (e) Differences in cell cycle distributions of TOV-21G transfected with si-HSP60 and control sequences, measured by FACS (*n* = 3). (f) The histogram of cell cycle distributions of TOV-21G transfected with si-HSP60 and control sequences, measured by FACS (*n* = 3). ^∗^*p* < 0.05, ^∗∗^*p* < 0.01, and ^∗∗∗^*p* < 0.001.

To explore the mechanism that si-HSP60 inhibited ovarian cancer cell proliferation, the apoptosis was measured by FACS between si-HSP60-ransfected TOV-21G and control TOV-21G that were stained with PI and annexin V. The results showed a higher ratio of cell apoptosis in the si-HSP60 transfection group compared to the control group (Figures 3(a) and 3(b)). Moreover, western blots for caspase-3, caspase-8, and caspase-9 were used to evaluate whether the extrinsic or intrinsic apoptosis pathway was activated. The results showed that caspase-3 and caspase-8 in the extrinsic apoptotic pathway were activated in HSP-silenced cancer cells (Figures 3(c) and 3(d)). Furthermore, the different distributions of the cell cycle were analyzed by FACS after transfection with si-HSP60. The results found that G0/G1 stage arrest was observed in the si-HSP60 transfection group compared to the control group; namely, the number of cells at the G0/G1 stage was increased and the number of cells at the S stage was decreased in the si-HSP60 group relative to the control group (Figures 3(e) and 3(f)). These data strongly demonstrated that HSP60 inhibited cell proliferation by blocking cell cycle progression and promoting its apoptosis.

### 3.4. HSP60 Might Regulate the Expressions or Folding of Important Proteins in Lipid Metabolism Pathways in Ovarian Cancers

**Figure 4:**
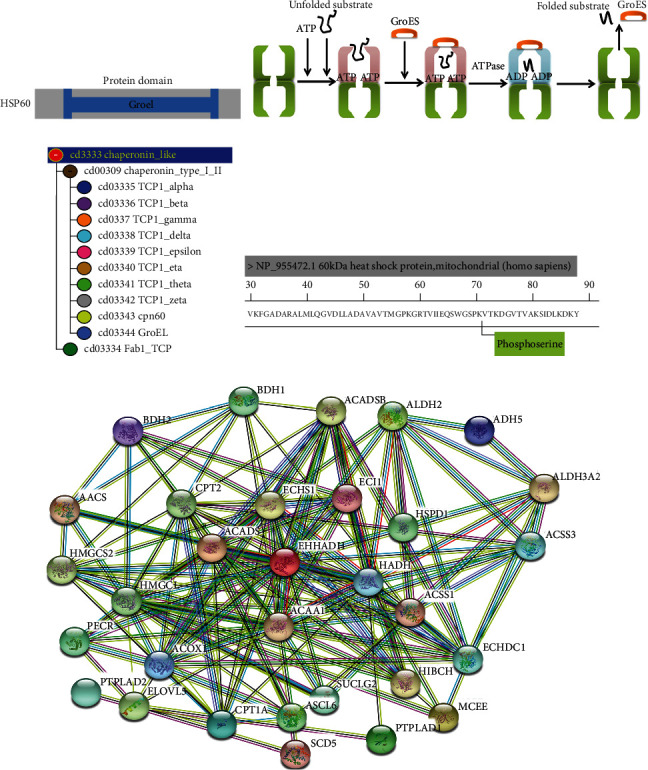
HSP60 associated with lipid metabolism pathways. (a) The drawing of the HSP60 protein domain. (b) The mechanism of mitochondrial protein folding within the HSP60-HSP10 complex. Modified from Horwich [56], with permission from Nature Publishers, copyright 2011. (c) Evolutionary relationship of the superfamily cl02777. CPN60 is the alias of HSP60. (d) HSP60 phosphorylation at residue Ser70. (e) Protein-protein interaction (PPI) in lipid metabolism pathways constructed with the STRING network. HSPD1 is the alias of HSP60.

HSP60 was reported to be used for survival prediction in advanced serous ovarian cancer [31]. Mitochondrial molecular chaperones like HSP60 regulated folding of mitochondrial matrix proteins, such as MCAD and ACADS. It provided a basis for characterization of mitochondria involved in folding and degradation of ACD proteins and lipid metabolism [29]. It was often considered that the HSP60 protein domain was similar to its prokaryotic ortholog GroEL (Figure 4(a)). The HSP10-HSP60 chaperoning protein folding complex played a role in protein folding. Briefly, unfolded proteins bound to the amino acid residue of HSP60 cavity. The mechanism of mitochondrial protein folding within the HSP60-HSP10 complex was investigated in Escherichia coli where the GroEL-GroES protein complex was exploited. After the substrate protein bound to the amino acid residue of HSP60 cavity, completed protein folding, and obtained its native conformation in several cycles, it would be released [11] (Figure 4(b)). Additionally, cd03344 (GroEL) was a member of the superfamily cl02777 (chaperonin_like), and the evolutionary relationship between cd03344 (GroEL) and cd03343 (HSP60) was near (Figure 4(c)). The iTRAQ quantitative proteomics identified the significantly upregulated expression of total HSP60 (fold change = 1.43, *p* = 3.81*E* − 2), and quantitative phosphoproteomics identified the significantly increased phosphorylation level at residue Ser70 in HSP60 (p-HSP60) (fold change = 2.34, *p* = 1.70*E* − 3), in ovarian cancer tissues relative to control ovary tissues (Figure 4(d)).

**Figure 5:**
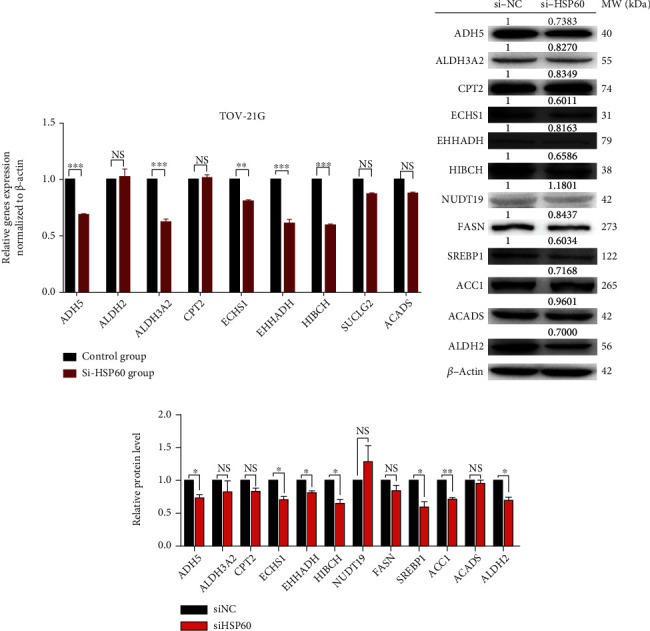
HSP60 regulation in lipid metabolism pathways. (a) The mRNA expressions of potential target genes of HSP60 were verified by qRT-PCR in ovarian cancer cells TO-21G with and without transfection with si-HSP60. (b) The protein expressions of potential target genes of HSP60 were verified by WB in ovarian cancer cells TO-21G with and without transfection with si-HSP60. (c) The histogram of protein expressions of potential target genes of HSP60 in ovarian cancer cells TO-21G with and without transfection with si-HSP60. ^∗^*p* < 0.05, ^∗∗^*p* < 0.01, and ^∗∗∗^*p* < 0.001.

The protein-protein interaction (PPI) network between the identified proteins in lipid metabolism pathways (Table 1) and HSP60 was constructed in ovarian cancer with the STRING network. This PPI network clearly demonstrated that HSP60 closely interacted with proteins HADH, ACAA1, ECHS1, ACADSB, ACOX1, PECR, CPT2, HMGCS2, HMGCL, BDH, CPT1A, ACADS, ECHDC1, ACSL6, ALDH3A2, ACSS1, ACSS3, ADH5, AACS, HIBCH, ECI1, SUCLG2, ALDH2, ELOVL5, HSPD1, PTPLAD2, EHHADH, MCEE, BDH2, SCD5, and PTPLAD1 in four lipid metabolism pathways, including fatty acid degradation, fatty acid metabolism, butanoate metabolism, and propanoate metabolism (Figure 4(e)), which indicated that HSP60 might play a role in the regulation of lipid metabolism. Further, after si-HSP60 or si-RNA negative control were transfected into TOV-21G cells, qRT-PCR analysis found that si-HSP60 significantly inhibited the expressions of potential target genes at the mRNA level when compared to controls, including ADH5 (fold change = 0.69, *p* < 0.001), ALDH3A2 (fold change = 0.62, *p* < 0.001), ECHS1 (fold change = 0.81, *p* = 0.0048), EHHADH (fold change = 0.61, *p* < 0.001), and HIBCH (fold change = 0.60, *p* < 0.001) in lipid metabolism pathways (Figure 5(a)). Moreover, western blot analysis found that si-HSP60 significantly inhibited the expressions of potential target genes in the protein level when compared to controls, including ADH5, ALDH2, ECHS1, EHHADH, and HIBCH in lipid metabolism pathways. Additionally, the key enzymes of fatty acid synthesis metabolism were also significantly expressed between si-HSP60 and control groups, including ACC1 and SREBP1 (Figures 5(b) and 5(c)).

**Figure 6:**
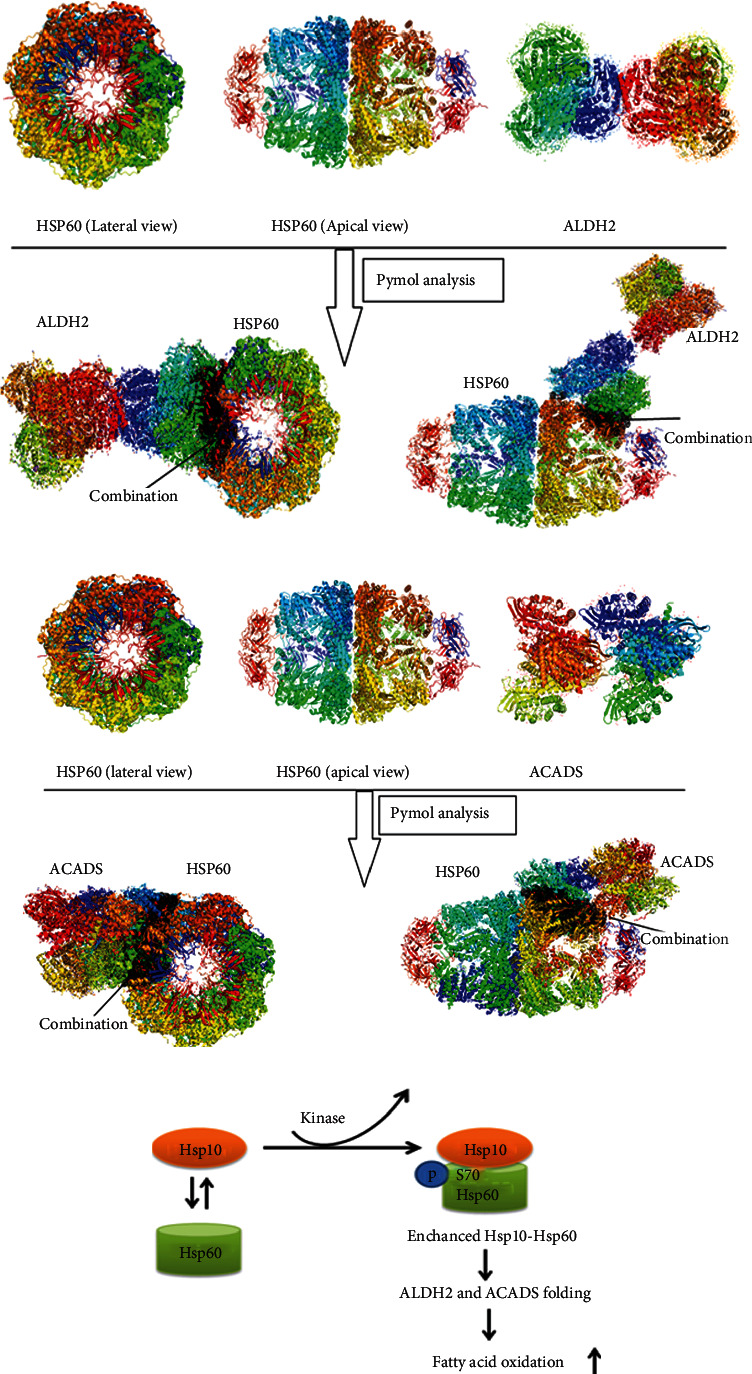
Spatial structural analysis of the molecular mechanism that HSP60 regulates lipid metabolism pathways. (a) HSP60-ALDH2 existed in binding sites with PYMOL based on PDB documents. (b) HSP60-ACADS existed in binding sites with PYMOL based on PDB documents. (c) The hypothesis that protein kinase phosphorylates HSP60, and the activated HSP60 in turn regulates protein folding of ALDH2 and ACADS in lipid metabolism pathways.

Even though some of these potential target genes (ALDH2, CPT2, SUCLG2, and ACADS) in the lipid metabolism pathway did not have mRNA expression changes in ovarian cancers, HSP60-regulated protein folding might be possible for those lipid metabolism-related proteins, based on the mechanism of the HSP10-HSP60 chaperoning protein folding complex. PDB documents of ALDH2, CPT2, SUCLG2, ACADS, and HSP60 were obtained from the RCSB PDB website (http://www.rcsb.org) and were used to construct the structures of human HSP60-ALDH2, HSP60-CPT2, HSP60-SUCLG2, and HSP60-ACADS complexes, with PYMOL. The results indicated that HSP60-ALDH2 and HSP60-ACADS might exist in binding sites (Hsp60:4PJ1, ALDH2:3N80, and ACADS:2VIG) (Figures 6(a) and 6(b)). ALDH2 participated in the metabolism or detoxification of various exogenous and endogenous aliphatic and aromatic aldehydes, which was a member of the aldehyde dehydrogenase 2 family and affected lipid peroxidation [32]. ACADS encoded a tetrameric mitochondrial flavoprotein, which was a member of the acyl-CoA dehydrogenase family. This enzyme catalyzed the initial step of the mitochondrial fatty acid beta-oxidation pathway [33].

**Figure 7:**
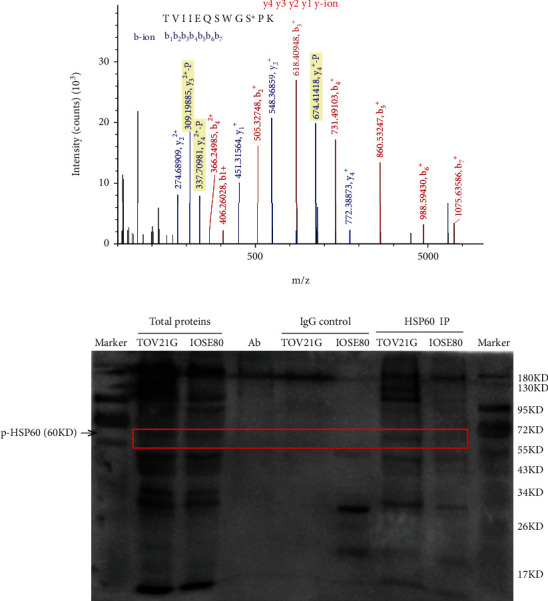
Analysis of HSP60 phosphorylation at residue Ser70 in human ovarian cancer tissue mitochondrial samples with mass spectrometry and in ovarian cancer cell TOV-21G with immunoprecipitation and western blot. (a) The MS/MS spectrum of phosphopeptide TVIIEQSWGS∗PK (S∗ = phosphoserine) derived from HSP60 in ovarian cancer tissues. (b) HSP60 was immunoprecipitated with an anti-HSP60 antibody, followed by phosphorylation analysis of immunoprecipitated HSP60 with western blot against the anti-phosphoserine antibody at residue Ser70 of HSP60 in ovarian cancer cells TOV-21G.

The LC-MS/MS spectrum from the phosphorylated peptide TVIIEQSWGS∗PK (*m*/*z* = 678.36865, S∗ = phosphorylated serine residue) derived from HSP60 (Swiss-Prot no. B3GQS7) was shown with a high-quality MS/MS spectrum, excellent signal-to-noise (S/N) ratio, and extensive product-ion *b*-ion and *y*-ion series (*b*_1_, *b*_2_, *b*_3_, *b*_4_, *b*_5_, *b*_6_, *b*_7_, *y*_1_, *y*_2_, *y*_3_, and *y*_4_) (Figure 7(a)). The phosphorylation site was localized at amino acid residue Ser70 in HSP60, and the phosphorylation level was significantly increased in ovarian cancers compared to controls (ratio of *T*/*N* = 2.34; *p* = 3.45*E* − 03). HSP60 immunoprecipitation in combination with a p-HSP60 immunoblot was used to semiquantify the p-HSP60 level in ovarian cancer cells TOV-21G, which was higher than control cells IOSE80 (Figure 7(b)). The immunoblot result was immune-based semiquantitative measurement and was consistent with rigorous MS/MS-based quantitative mitochondrial phosphoproteome data in ovarian cancers. Thereby, we assume that protein kinase phosphorylates HSP60, and the activated HSP60 in turn regulates protein folding of ALDH2 and ACADS to change lipid metabolism pathways (Figure 6(c)).

## 4. Discussion

During the last decade, a great attention has been focused on metabolic alterations of cancer. Studies on “Warburg effect” and “the reverse Warburg effect” have set the pioneering work of glycometabolism functions in cancer [34]. Despite metabolic plasticity being one of the hallmarks described in human disease, the process of glycometabolism disorder was considered conditioned and inefficient for neoplastic transformation. Lipid metabolic reprogramming is less known. Lipid metabolism disorders are associated with an increase in plasma lipids such as low-density lipoprotein, very low-density lipoprotein, and triglycerides which most commonly lead to cardiovascular diseases. They are recently becoming more important in the peculiarities of tumor cell metabolism [35]. Interestingly, the results demonstrated that ovarian cancer cells exhibit an increased dependence on lipid metabolism, such as biosynthesis of unsaturated fatty acids, butanoate metabolism, fatty acid degradation, fatty acid metabolism, propanoate metabolism, and peroxisome pathway, which might play an important role in invasion and metastasis of ovarian cancer. These findings are in agreement with those obtained from other literature. For instance, lipids located in cell membranes (particularly cholesterol and sphingolipids) form lipid rafts, which contain an array of signaling proteins and receptors involved in the cancer-relevant signaling pathways [36]. One study revealed that upregulation of genes (ABCA1, ACSL1, AGPAT1, and SCD) related to lipogenesis and cholesterol synthesis pathways was associated with poor survival outcomes in colorectal cancer patients [19]. Indeed, the coculture of adipocytes and ovarian cancer cells results in promoting ovarian cancer cell growth [22]. The Cancer Genome Atlas provides a system view of metabolic heterogeneity (including lipid metabolism) within and across cancer types and identifies pathway cross-talk, suggesting related predictive, therapeutic, and prognostic utilities [35]. Moreover, the present study on the basis of lipid metabolism in ovarian cancer suggests potential biomarkers, including BDH2, ADH5, PRKCDBP, ECHS1, ACOX1, BDH1, NUDT19, PXMP4, PECR, HIBCH, PEX13, EHHADH, ECI1, ECHDC1, SUCLG2, ABCD3, PHYH, PEX14, IDH2, CPT2, GSTK1, HMGCS2, and ACAA1 in lipid metabolism pathways. Some of them have been reported as biomarkers in other types of cancers, such as GSTK1, IDH2, CAVIN3, HMGCS2, and ACOX1. GSTK1, which is known as a tumor marker, showed some kind of relationship with cancer cell proliferation [37]. In fact, GSTK1 was predominantly expressed in many types of tumor cells and regarded as its marker protein, including oral premalignant and malignant lesions [38] and human erythroleukemia [39]. IDH2 was also reported as a diagnostic and prognostic serum biomarker for non-small-cell lung cancer [40]. Lipid metabolism was closely associated with the occurrence and development of cancer. Therapeutic targeting in lipid metabolism pathways or key related molecules would be challenges and opportunities for ovarian cancer treatment.

HSPs, stimulated by heat shock or other stressors, constitute a large protein family implicated in protein folding and maturation. HSPs have an impact on cellular proliferation, differentiation, and carcinogenesis, whose major family members include HSP27, HSP60, HSP10, HSP40, HSP90, HSP70, and large HSPs. A review article addressed the comprehensive roles of major HSPs in cancer development and pharmacology, which summarized HSP family members in cancer progression, metastasis, diagnosis, treatment, and drug resistance [11]. For instance, HSP27 was identified as phosphorylated at residues Ser15, Ser78, and Ser82, which might regulate the functions of human HSP27, including regulating p53 signaling, inducing the expression of phosphatase and tensin homolog (PTEN), and interaction with cytochrome c [41]. High expression of multiple HSP40 family members was observed in gastric cancer, lung cancer, colorectal cancer, and cervical cancer, and HSP40 had dual roles in both procancer processes and anticancer [42]. This present study detected the expression of total HSP60 and phosphorylated HSP60 in ovarian cancer and control tissues, which showed that total HSP60 and phosphorylated HSP60 were both upregulated in ovarian cancers. In cells with increased or phosphorylated HSP60 levels, the amounts of total mitochondrial short-chain acyl-CoA dehydrogenase (ACADS) proteins and folded ACADS were increased, which might influence mitochondrial protein folding and lipid metabolism. One speculated that HSP60 was involved in the folding of ACADS variant proteins and alerting ACADS enzyme activity, which in turn regulated the lipid metabolism pathway and fatty acid oxidation. HSP60 was mainly located in the mitochondria where it combined as the HSP60/HSP10 complex [43]. Moreover, HSP60 not only was located in mitochondria but also has been found widespread in the body, such as in the cytosol, extracellular space, cell surface, and peripheral blood [44]. Overexpression of HSP60 proved poor prognosis in various carcinomas, including advanced ovarian cancer, head and neck cancer, non-small-cell lung cancer, prostate cancer, gastric cancer, neuroblastoma, bronchial cancer, and colorectal cancer [11]. Moreover, increased HSP60 expression was correlated with chemotherapy drug resistance, and inhibition of HSP60 expression somehow reversed drug resistance [45]. Various mechanisms of HSP60 associated with procarcinogenesis were reported, including inhibiting intracellular protein clusterin, interacting with cyclophilin D, and being involved in the protein nuclear factor-kB pathway [11]. The most exciting feature was that a variety of anticancer drugs were approved by the FDA to target HSPs for cancer treatment, such as sorafenib (Nexavar) [46] and Ruxolitinib (Jakafi) [47]. Overall, our findings were consistent with previous researches, and it was the first time to integrate quantitative mitochondrial proteomics and quantitative mitochondrial phosphoproteomics in human ovarian cancers to explore the relationship between HSP60 and lipid metabolism.

PTM is one of the most common forms in living cells and is a common reason for the formation of human proteoforms [48]. Approximately thirty percent proteins encoded were evaluated to be phosphorylated during their biological cycle [49]. Protein phosphorylation was a reversible process regulating cellular biology characteristics such as cell proliferation, division, migration, and differentiation through highly dynamic or signaling pathways containing phosphorylase activity [50]. So much evidence had reported that various phosphorylation phenotypes induced different pathological features. Researchers had investigated the different phosphorylation level in cancer cells, discussing its correlation with clinicopathologic features and prognosis of carcinoma. Phosphorylation was associated with histopathology grade and the depth of tissue invasion [51]. Mass spectrometry has been widely used to identify and quantify protein PTMs in specific pathways and proteomes [52]. However, few phosphoproteomic approaches detected the subcellular localization of phosphoproteins, which was a significant factor to understand the characterization of the subcellular phosphoproteome in regulating biological processes [53]. The increasing number of identified mitochondrial phosphoproteins, phosphatases, and kinases in recent years suggested that reversible protein phosphorylation played an important part in the control of mitochondrial processes. In addition, many mitochondrial phosphoproteins probably still remain to be identified. HSP60 was also phosphorylated at tyrosine residue (Tyr_227_) by an activated form of Src kinase, which correlated with proteasomal degradation of HSP60. Phosphorylation and subsequent transient degradation of mitochondrial HSP60 resulted in the inhibition of premature import of nonstructural protein into mitochondria, thereby delaying early apoptosis [54]. HSP60 phosphorylation might not only influence the function or molecular conformation of itself but also regulate other protein folding or cellular protein movement. HSP60 functioned in the regulation of the cellular process in various types of cancer cells to influence tissue growth, which could serve as a target for anticancer therapy [55]. It might be effective to develop specific locus targeting HSP60 in ovarian cancers.

## 5. Conclusion

Abnormal ROS metabolism was significantly found in ovarian cancers based on mitochondrial proteomic data. The phosphorylation regulation of HSP60 that influences lipid metabolism demonstrated molecular mechanisms of oxidative stress-related diseases. Understanding the mechanisms that cause differences in HSP60 expression levels in ovarian cancer may be useful in the development of therapeutic approaches for pharmaceutical, clinical, and biotechnological applications. The present study integrated a large-scale quantitative mitochondrial proteomic data and quantitative mitochondrial phosphoproteomic data, which found a certain number of proteins with the potential biomarkers, drug targets, and a novel vision in the lipid metabolism biomechanism of human ovarian cancer. Further studies would be helpful to identify new biomarkers and potential drug targets of ovarian cancer.

## Figures and Tables

**Table 1:** KEGG pathway analysis of 5115 mtEPs revealed four statistically significant lipid metabolism pathway alterations in ovarian cancer.

Lipid metabolism pathways	Accession	Description	Gene name	Coverage	Unique peptides	Peptides	PSMs	MW (kDa)	Calc. pI	Average tumor/control	*t*-test *p* value
Fatty acid degradation pathway	E7ERD7	Long-chain fatty acid-CoA ligase 6 OS=Homo sapiens GN=ACSL6 PE=1 SV=1—[E7ERD7_HUMAN]	ACSL6	8.27	3	5	7	75.20	7.42	1.38	6.22*E* − 03
	B2RAQ8	cDNA, FLJ95058, highly similar to Homo sapiens carnitine palmitoyltransferase 1A (liver) (CPT1A), nuclear gene encoding mitochondrial protein, mRNA OS=Homo sapiens PE=2 SV=1—[B2RAQ8_HUMAN]	CPT1A	34.41	22	22	47	88.28	8.59	1.41	9.89*E* − 03
	P23786	Carnitine O-palmitoyltransferase 2, mitochondrial OS=Homo sapiens GN=CPT2 PE=1 SV=2—[CPT2_HUMAN]	CPT2	42.40	5	24	77	73.73	8.18	2.05	1.99*E* − 02
	A0A024R8L7	Acyl-coenzyme A oxidase OS=Homo sapiens GN=ACOX1 PE=3 SV=1—[A0A024R8L7_HUMAN]	ACOX1	29.85	14	14	23	74.64	7.62	1.53	3.40*E* − 02
	D4QEZ8	Short-chain acyl-CoA dehydrogenase OS=Homo sapiens GN=ACADS PE=2 SV=1—[D4QEZ8_HUMAN]	ACADS	27.67	8	8	19	44.33	7.72	1.33	6.43*E* − 06
	P45954	Short/branched chain-specific acyl-CoA dehydrogenase, mitochondrial OS=Homo sapiens GN=ACADSB PE=1 SV=1—[ACDSB_HUMAN]	ACADSB	25.93	8	8	15	47.46	6.99	1.29	1.06*E* − 02
	Q08426	Peroxisomal bifunctional enzyme OS=Homo sapiens GN=EHHADH PE=1 SV=3—[ECHP_HUMAN]	EHHADH	33.47	22	22	34	79.44	9.14	1.62	2.25*E* − 03
	P30084	Enoyl-CoA hydratase, mitochondrial OS=Homo sapiens GN=ECHS1 PE=1 SV=4—[ECHM_HUMAN]	ECHS1	75.86	19	19	156	31.37	8.07	1.52	3.56*E* − 03
	Q08426	Peroxisomal bifunctional enzyme OS=Homo sapiens GN=EHHADH PE=1 SV=3—[ECHP_HUMAN]	EHHADH	33.47	22	22	34	79.44	9.14	1.62	2.25*E* − 03

Fatty acid metabolism pathway	Q9P035	Very-long-chain (3R)-3-hydroxyacyl-CoA dehydratase 3 OS=Homo sapiens GN=HACD3 PE=1 SV=2—[HACD3_HUMAN]	HACD3	22.93	7	7	26	43.13	8.94	1.06	6.65*E* − 2
	Q5VWC8	Very long-chain (3R)-3-hydroxyacyl-CoA dehydratase 4 OS=Homo sapiens GN=HACD4 PE=1 SV=1—[HACD4_HUMAN]	HACD4	4.31	2	2	3	27.50	8.57	1.26	1.74*E* − 02
	A0A0A0MTI6	Elongation of very long-chain fatty acid protein OS=Homo sapiens GN=ELOVL5 PE=1 SV=1—[A0A0A0MTI6_HUMAN]	ELOVL5	3.05	1	1	2	30.72	7.52	1.37	1.94*E* − 03
	P09110	3-Ketoacyl-CoA thiolase, peroxisomal OS=Homo sapiens GN=ACAA1 PE=1 SV=2—[THIK_HUMAN]	ACAA1	46.23	4	14	50	44.26	8.44	2.70	1.00*E* − 02
	D4QEZ8	Short-chain acyl-CoA dehydrogenase OS=Homo sapiens GN=ACADS PE=2 SV=1—[D4QEZ8_HUMAN]	ACADS	27.67	8	8	19	44.33	7.72	1.33	6.43*E* − 06
	P45954	Short/branched chain-specific acyl-CoA dehydrogenase, mitochondrial OS=Homo sapiens GN=ACADSB PE=1 SV=1—[ACDSB_HUMAN]	ACDSB	25.93	8	8	15	47.46	6.99	1.29	1.06*E* − 02
	A0A024R8L7	Acyl-coenzyme A oxidase OS=Homo sapiens GN=ACOX1 PE=3 SV=1—[A0A024R8L7_HUMAN]	ACOX1	29.85	14	14	23	74.64	7.62	1.53	3.40*E* − 02
	E7ERD7	Long-chain fatty acid-CoA ligase 6 OS=Homo sapiens GN=ACSL6 PE=1 SV=1—[E7ERD7_HUMAN]	E7ERD7	8.27	3	5	7	75.20	7.41	1.38	6.22*E* − 03
	B2RAQ8	cDNA, FLJ95058, highly similar to Homo sapiens carnitine palmitoyltransferase 1A (liver) (CPT1A), nuclear gene encoding mitochondrial protein, mRNA OS=Homo sapiens PE=2 SV=1—[B2RAQ8_HUMAN]	CPT1A	34.41	22	22	47	88.28	8.59	1.41	9.89*E* − 03
	P23786	Carnitine O-palmitoyltransferase 2, mitochondrial OS=Homo sapiens GN=CPT2 PE=1 SV=2—[CPT2_HUMAN]	CPT2	42.40	5	24	77	73.73	8.18	2.05	1.99*E* − 02
	Q08426	Peroxisomal bifunctional enzyme OS=Homo sapiens GN=EHHADH PE=1 SV=3—[ECHP_HUMAN]	EHHADH	33.47	22	22	34	79.44	9.14	1.62	2.25*E* − 03
	P30084	Enoyl-CoA hydratase, mitochondrial OS=Homo sapiens GN=ECHS1 PE=1 SV=4—[ECHM_HUMAN]	ECHM	75.86	19	19	156	31.37	8.07	1.52	3.56*E* − 03
	Q16836	Hydroxyacyl-coenzyme A dehydrogenase, mitochondrial OS=Homo sapiens GN=HADH PE=1 SV=3—[HCDH_HUMAN]	HCDH	64.01	16	16	69	34.27	8.85	1.34	2.11*E* − 02
	Q8N8R3	Mitochondrial basic amino acid transporter OS=Homo sapiens GN=SLC25A29 PE=1 SV=2—[MCATL_HUMAN]	MCATL	20.13	6	6	7	32.04	8.75	1.07	4.30*E* − 02
	Q9BY49	Peroxisomal trans-2-enoyl-CoA reductase OS=Homo sapiens GN=PECR PE=1 SV=2—[PECR_HUMAN]	PECR	26.40	7	7	10	32.52	8.81	1.57	4.14*E* − 04
	Q86SK9	Stearoyl-CoA desaturase 5 OS=Homo sapiens GN=SCD5 PE=1 SV=2—[SCD5_HUMAN]	SCD5	9.70	2	2	4	37.59	9.61	0.77	1.05*E* − 02

Butanoate metabolism pathway	D4QEZ8	Short-chain acyl-CoA dehydrogenase OS=Homo sapiens GN=ACADS PE=2 SV=1—[D4QEZ8_HUMAN]	ACADS	27.67	8	8	19	44.33	7.72	1.33	6.43*E* − 06
	Q08426	Peroxisomal bifunctional enzyme OS=Homo sapiens GN=EHHADH PE=1 SV=3—[ECHP_HUMAN]	EHHADH	33.47	22	22	34	79.44	9.14	1.62	2.25*E* − 03
	P30084	Enoyl-CoA hydratase, mitochondrial OS=Homo sapiens GN=ECHS1 PE=1 SV=4—[ECHM_HUMAN]	ECHS1	75.86	19	19	156	31.37	8.07	1.52	3.56*E* − 03
	Q16836	Hydroxyacyl-coenzyme A dehydrogenase, mitochondrial OS=Homo sapiens GN=HADH PE=1 SV=3—[HCDH_HUMAN]	HADH	64.01	16	16	69	34.27	8.85	1.34	2.11*E* − 02
	Q08426	Peroxisomal bifunctional enzyme OS=Homo sapiens GN=EHHADH PE=1 SV=3—[ECHP_HUMAN]	EHHADH	33.47	22	22	34	79.44	9.14	1.62	2.25*E* − 03
	Q02338	D-Beta-hydroxybutyrate dehydrogenase, mitochondrial OS=Homo sapiens GN=BDH1 PE=1 SV=3—[BDH_HUMAN]	BDH1	41.98	11	11	23	38.13	8.95	1.54	2.99*E* − 03
	Q9BUT1	3-Hydroxybutyrate dehydrogenase type 2 OS=Homo sapiens GN=BDH2 PE=1 SV=2—[BDH2_HUMAN]	BDH2	17.14	4	4	6	26.71	7.65	0.51	1.81*E* − 04
	Q86V21	Acetoacetyl-CoA synthetase OS=Homo sapiens GN=AACS PE=1 SV=1—[AACS_HUMAN]	AACS	2.68	2	2	2	75.10	6.24	1.35	5.30*E* − 04
	P54868	Hydroxymethylglutaryl-CoA synthase, mitochondrial OS=Homo sapiens GN=HMGCS2 PE=1 SV=1—[HMCS2_HUMAN]	HMGCS2	12.80	7	7	11	56.60	8.16	2.17	2.10*E* − 03
	B1AK13	3-Hydroxymethyl-3-methylglutaryl-coenzyme A lyase (hydroxymethylglutaricaciduria), isoform CRA_b OS=Homo sapiens GN=HMGCL PE=2 SV=1—[B1AK13_HUMAN]	HMGCL	53.00	13	14	49	31.71	7.61	1.33	2.91*E* − 03

Propanoate metabolism pathway	Q7Z5G3	Acetyl-coenzyme A synthetase OS=Homo sapiens GN=ACSS1 PE=2 SV=1—[Q7Z5G3_HUMAN]	ACSS1	17.32	10	10	11	74.61	7.23	0.82	1.17*E* − 02
	A0A0B4J1R2	Acyl-CoA synthetase short-chain family member 3, mitochondrial OS=Homo sapiens GN=ACSS3 PE=1 SV=1—[A0A0B4J1R2_HUMAN]	ACSS3	42.48	23	23	62	74.54	8.63	0.83	8.45*E* − 04
	Q6NVY1	3-Hydroxyisobutyryl-CoA hydrolase, mitochondrial OS=Homo sapiens GN=HIBCH PE=1 SV=2—[HIBCH_HUMAN]	HIBCH	34.46	14	14	29	43.45	8.19	1.58	2.50*E* − 03
	Q08426	Peroxisomal bifunctional enzyme OS=Homo sapiens GN=EHHADH PE=1 SV=3—[ECHP_HUMAN]	EHHADH	33.47	22	22	34	79.44	9.14	1.62	2.25*E* − 03
	P30084	Enoyl-CoA hydratase, mitochondrial OS=Homo sapiens GN=ECHS1 PE=1 SV=4—[ECHM_HUMAN]	ECHS1	75.86	19	19	156	31.37	8.07	1.52	3.56*E* − 03
	Q9NTX5	Ethylmalonyl-CoA decarboxylase OS=Homo sapiens GN=ECHDC1 PE=1 SV=2—[ECHD1_HUMAN]	ECHDC1	9.45	3	3	10	33.68	8.21	1.69	3.67*E* − 03
	Q96I99	Succinate-CoA ligase (GDP-forming) subunit beta, mitochondrial OS=Homo sapiens GN=SUCLG2 PE=1 SV=2—[SUCB2_HUMAN]	SUCLG2	44.91	19	20	115	46.48	6.39	1.71	8.17*E* − 04
	Q96I99	Succinate-CoA ligase (GDP-forming) subunit beta, mitochondrial OS=Homo sapiens GN=SUCLG2 PE=1 SV=2—[SUCB2_HUMAN]	SUCLG2	44.91	19	20	115	46.48	6.39	1.71	8.17*E* − 04
	Q96PE7	Methylmalonyl-CoA epimerase, mitochondrial OS=Homo sapiens GN=MCEE PE=1 SV=1—[MCEE_HUMAN]	MCEE	38.07	5	5	8	18.74	9.09	1.22	4.87*E* − 02

## Data Availability

All the data used in this study are collected in this article and supplemental materials.

## Abbreviations

ACADS: Acyl-CoA dehydrogenase
ESI: Electrospray ionization
FACS: Fluorescence-activated cell sorting
HSL: Hormone-sensitive lipase
HSPs: Heat shock proteins
IPA: Ingenuity pathway analysis
iTRAQ: Isobaric tags for relative and absolute quantitation
KEGG: Kyoto Encyclopedia of Genes and Genomes
LMF: Lipid-mobilizing factor
MCAD: Medium-chain acyl-CoA dehydrogenase
MS: Mass spectrometry
mtDEPs: Mitochondrial differentially expressed proteins
mtEPs: Mitochondrial expressed proteins
OC: Ovarian cancer
PPI: Protein-protein interaction
PTMs: Posttranslational modifications
qRT-PCR: Quantitative real-time PCR
ROS: Reactive oxygen species.

## Supplementary Materials

Supplementary 1 Supplemental Table 1: the list of RNA molecules that have been assessed on the cell lines.

Supplementary 2 Supplementary Figure 1: KEGG pathway analysis of 5115 ovarian cancer mitochondrial proteins (mtEPs) revealed four lipid metabolism pathway alterations in human ovarian cancers.
