# Supplementary material for: HSP60 Regulates Lipid Metabolism in Human Ovarian Cancer
Source: Oxid Med Cell Longev. 2021 Sep 12;2021:6610529. doi: 10.1155/2021/6610529 (PMC8452972; doi:10.1155/2021/6610529)
Supplement: Supplementary 1 — Supplemental Table 1: the list of RNA molecules that have been assessed on the cell lines. [file 6610529.f1.doc]

**Supplemental Table 1: The list of RNA molecules that have been assessed on the cell lines.** Note: F = forward, and R = reverse.

| **RNA type** | **Primer name** | **Primer sequence (from 5' to 3')** |
| --- | --- | --- |
| **Reference gene** | β-actin-F | AGGGGCCGGACTCGTCATACT |
|  | β-actin-R | GGCGGCACCACCATGTACCCT |
| **mRNA** | ADH5-F | GCAGTTATCATGGGCTGTAAAG |
|  | ADH5-R | TGCTCTCATGACCTTCACATTA |
|  | ALDH2-F | TCAGTTTAAGAAGATCCTCGGC |
|  | ALDH2-R | TCTTGAACTTCAGGATCTGCAT |
|  | ALDH3A2-F | AGGAAGTCATTACTGTCCTTGG |
|  | ALDH3A2-R | GCACGTTCTTCTTAACTGGTTT |
|  | CPT2-F | GTGATGGTGTGGCAGTGCTCAG |
|  | CPT2-R | TGGCTGGCTCTGTGGAGTGAC |
|  | ECHS1-F | GTGATATCATCTATGCCGGTGA |
|  | ECHS1-R | GTCTCAACAGGACAAATCTTGC |
|  | EHHADH-F | CTCAAGCTGGGCATTCTAGATA |
|  | EHHADH-R | GTCTACGGGATTCTAGAGGTTG |
|  | HIBCH-F | TGGAGGCTCATGTCGAGGTT |
|  | HIBCH-R | GCTGCATCTGTGTGCTTGGA |
|  | SUCLG2-F | AAGTTAACAAGGTGATGGTTGC |
|  | SUCLG2-R | TTAAAAATGAGCTCCGGGTTTG |
|  | ACADS-F | CAGTTACACACCATCTACCAGT |
|  | ACADS-R | GCTGGGAAGAGATGTTCCTTAT |
