# Supplementary material for: HSP60 Regulates Lipid Metabolism in Human Ovarian Cancer
Source: Oxid Med Cell Longev. 2021 Sep 12;2021:6610529. doi: 10.1155/2021/6610529 (PMC8452972; doi:10.1155/2021/6610529)

**Supplementary Figure 1**

**KEGG pathway analysis of 5115 ovarain cancer mitochondrial proteins (mtEPs) revealed four lipid metabolism pathway alterations in human ovarian cancers.**

**(i) Fatty acid degradation [24]**

**(ii) Fatty acid metabolism**

**(iii) Butanoate metabolism**

**(iv) propanoate metabolism, in human ovarian cancer tissues.**

**(i) Fatty acid degradation**


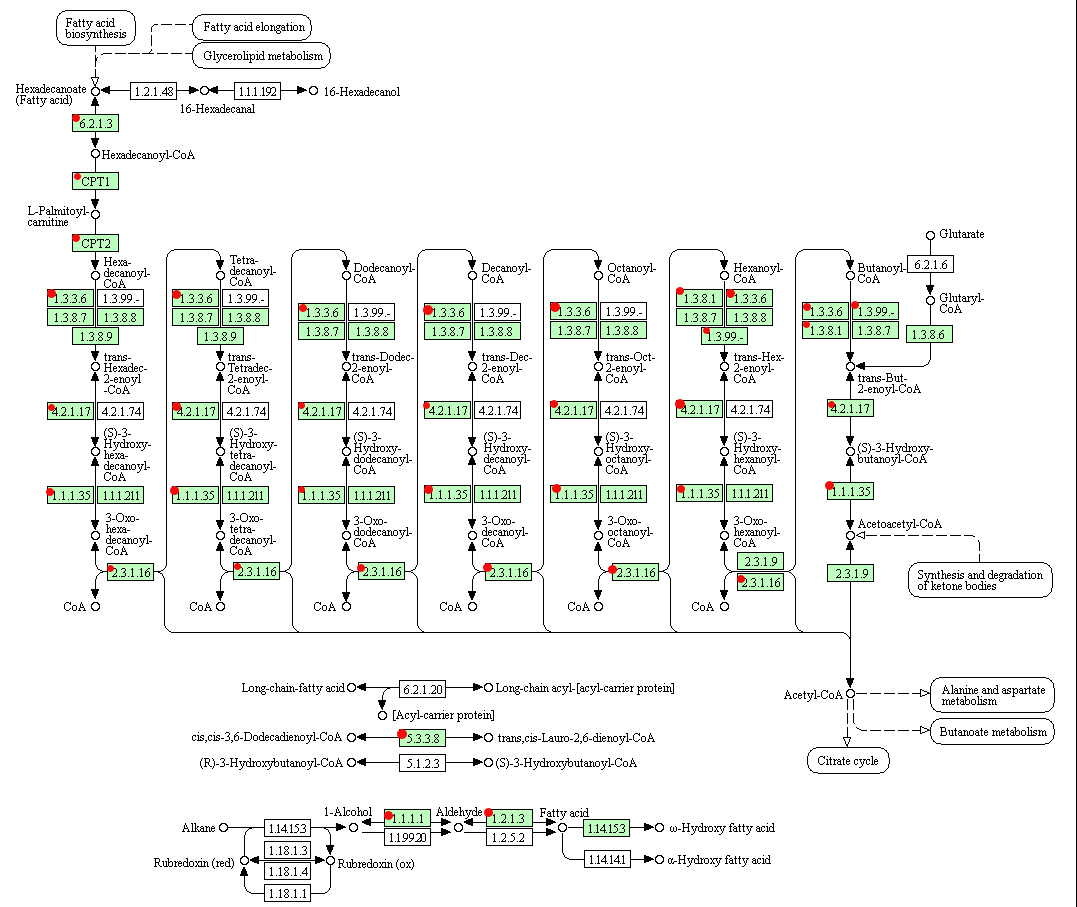


**(ii) Fatty acid metabolism**


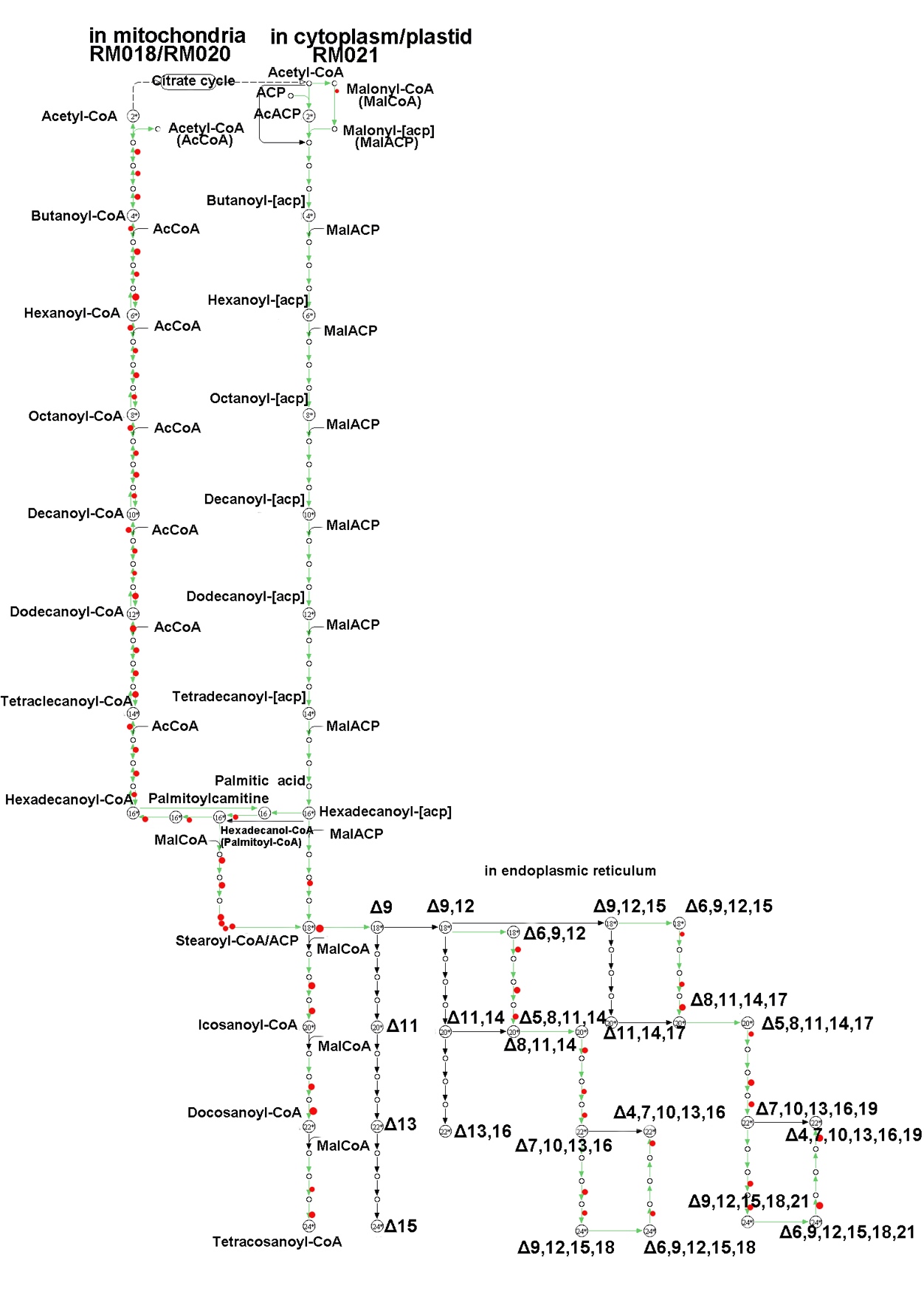


**(iii) Butanoate metabolism**


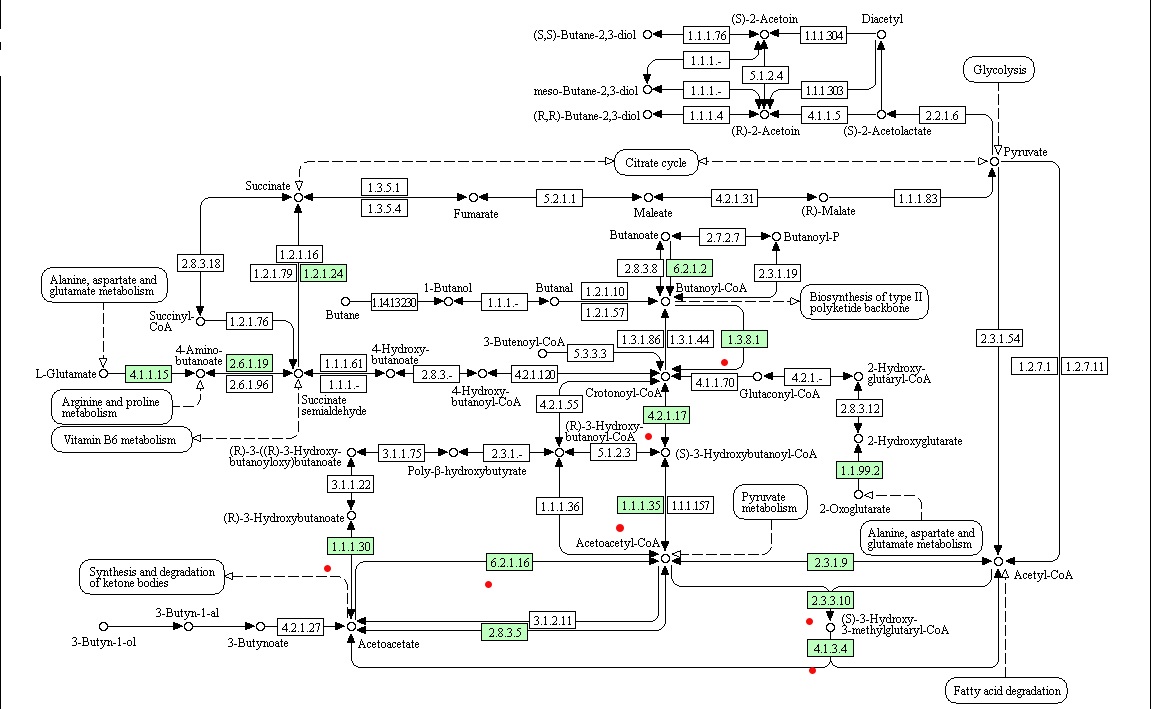


**(iv) propanoate metabolism**


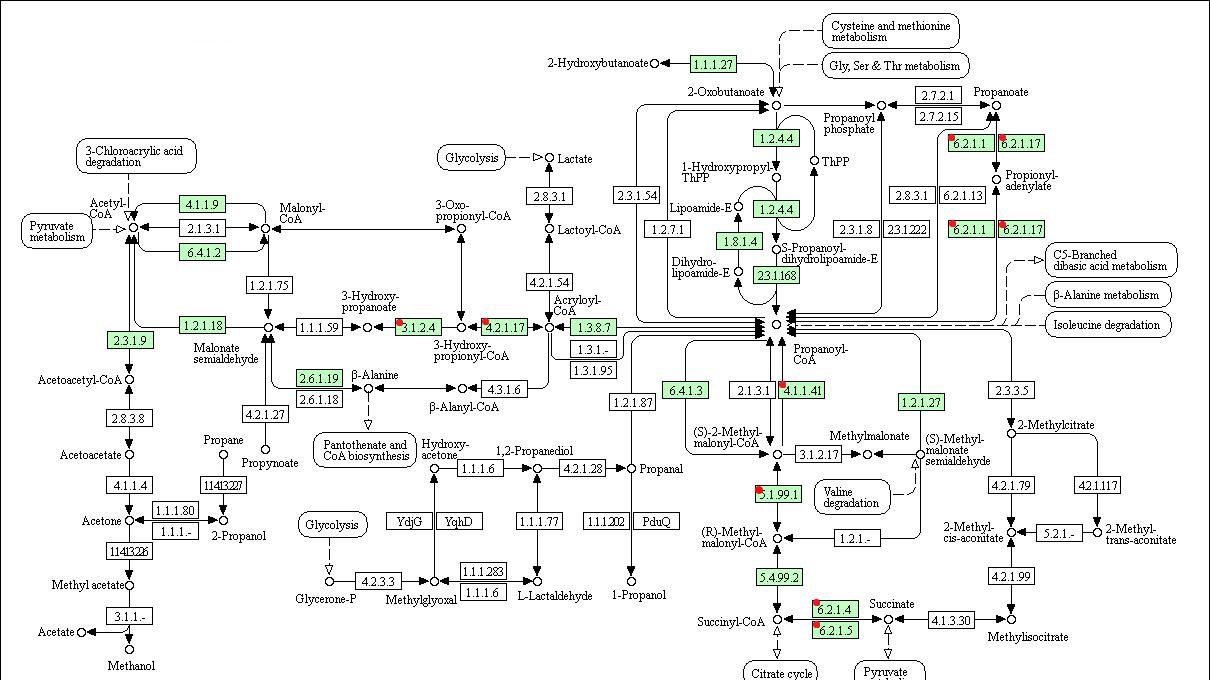

Supplement: Supplementary 2 — Supplementary Figure 1: KEGG pathway analysis of 5115 ovarian cancer mitochondrial proteins (mtEPs) revealed four lipid metabolism pathway alterations in human ovarian cancers. [file 6610529.f2.docx]
